# Testing Multi-Theory Model (MTM) in Explaining Sunscreen Use among Florida Residents: An Integrative Approach for Sun Protection

**DOI:** 10.3390/healthcare9101343

**Published:** 2021-10-10

**Authors:** Manoj Sharma, Matthew Asare, Erin Largo-Wight, Julie Merten, Mike Binder, Ram Lakhan, Kavita Batra

**Affiliations:** 1 Department of Social and Behavioral Health, School of Public Health, University of Nevada, Las Vegas, NV 89119, USA; manoj.sharma@unlv.edu; 2 Department of Public Health, Baylor University, Waco, TX 76798, USA; matt_asare@baylor.edu; 3 Department of Public Health, University of North Florida, Jacksonville, FL 32224, USA; largo.wight@unf.edu (E.L.-W.); jmerten@unf.edu (J.M.); 4 Institute of Environmental Research and Education, University of North Florida, Jacksonville, FL 32224, USA; 5 Department of Political Science and Public Administration, University of North Florida, Jacksonville, FL 32224, USA; m.binder@unf.edu; 6 Department of Health and Human Performance, Berea College, Berea, KY 40404, USA; Lakhanr@berea.edu; 7 Office of Research, Kirk Kerkorian School of Medicine, University of Nevada, Las Vegas, NV 89102, USA

**Keywords:** multi-theory model, skin cancer, sunscreen, Florida, integrative medicine

## Abstract

Florida residents have the second highest incidence of skin cancer in the nation. Sunscreen usage was found to be the one of the most effective integrative health approaches for reducing risk of skin cancer. Given the limited information on the likelihood of adopting and continuing sunscreen usage behavior, this cross-sectional study aimed to examine the correlates of initiating and sustaining sunscreen usage behavior among Florida dwellers, using the fourth-generation, multi-theory model (MTM) of behavior change. A web-based survey containing 51 questions was emailed to Florida residents aged 18 years or above, who were randomly selected from the state voter file. Psychometric validity of the survey instrument was established using structural equation modeling, and Cronbach’s alpha values were calculated for assessing the internal consistency. An independent-samples-*t*-test and hierarchical multiple regression tests were used to analyze the data. The results indicated that participants who engaged in sunscreen usage behavior, participatory dialogue (β = 0.062, *p* < 0.05), behavioral confidence (β = 0.636, *p* < 0.001), and changes in the physical environment (β = 0.210, *p* < 0.001) were statistically significant and accounted for 73.6% of the variance in initiating sunscreen usage behavior. In addition, the constructs of emotional transformation (β = 0.486, *p* < 0.001) and practice for change (β = 0.211, *p* < 0.001), as well as changes in the social environment (β = 0.148, *p* < 0.001) were significant predictors of maintaining sunscreen usage behavior and contributed to 59% of variance in sustenance. These findings offer a valuable insight regarding the applicability of MTM models to guiding public health interventions promoting sunscreen usage and preventing UV radiation risk and related skin cancer.

## 1. Introduction

Sunscreen offers an integrative health approach to sun protection, to prevent skin cancers. Skin cancer is among the most common forms of cancers in the United States (U.S.), affecting nearly 10,000 people every day [1]. Over three million cases of non-melanoma skin cancers (NMSC), including basal cell carcinoma (BCC) and squamous cell carcinoma (SCC), are diagnosed annually, with one in five Americans projected to develop the cancer during their lifetime [2]. The incidence rates may vary across states and regions, depending upon sociodemographic and environmental factors, and rates of cancer screening [1,3]. Florida has the second highest incidence rate of skin cancers in the U.S., which can be partly explained by its high ultraviolet (UV) index [3,4,5]. Over 600 Floridians die of skin cancer each year, and this mortality rate has doubled over the past few decades [3]. These rates are also underestimated, due to lack of NMSC reporting in cancer registries [1,6]. However, a report of the Medical Expenditure Panel Survey indicated that nearly 4.3 million people were treated for NMSC in 2015 in the U.S. [6]. Collective evidence suggests an increase in the national, as well as global, incidence of NMSC compared to other forms of non-preventable cancers combined [1,7,8,9,10].

Given the continued increase in incidence, the healthcare cost associated with skin cancer is substantial, making it the fifth most expensive disease in the U.S. [1,11]. Nearly five million people have been treated for some form of skin cancer, which cost the nation over eight billion dollars [4]. This underscores the need for adopting and reinforcing cost-effective yet simple preventive strategies, especially sunscreen use, which is the single most modifiable risk factor of skin cancer, other than avoiding ultraviolet (UV) exposure [2,12].

Other risk factors for skin cancers include old age, race, family history, male gender, long-term skin inflammation, and immunocompromised status [7,13,14]. NMSC occurs more often in white people than people of color, due to lower melanin (photo-protective pigment) production in the former group [14]. However, the worst prognosis was noted among people of color [15,16]. According to the previous reports, incidence and mortality associated with NMSC among people of color may be underestimated, given the scarcity of data [14]. UV radiation from the sun or indoor tanning machines has been directly associated with the development of skin cancer [12]. The risk of skin cancer can be significantly reduced by limiting sun exposure [12]. The American Cancer Society (ACS) recommends avoiding the sun during peak hours (10 am–4 pm), seeking shade when outdoors, wearing sun protective clothing, including sunglasses and a wide-brimmed hat, and frequently applying sunscreen (SPF > 30) with both UVA and UVB (broadband) protection [17].

Proper sunscreen use has been linked to a reduction in squamous cell and malignant melanoma skin cancer development, by 40% and 50%, respectively [18,19]. The best method of preventing skin cancer in the population is to increase sunscreen usage in the community, to protect skin from harmful UV radiation exposure. Regrettably, the utilization of sunscreen is low despite the well-established protective benefits of sunscreen in preventing skin cancers [18,19]. Personal barriers (dislike of the appearance or feel of sunscreen), time constraints, and economic barriers were commonly cited contributing factors to sunscreen underuse [20]. According to the 2015 National Health Interview Survey-Cancer Control Supplement analysis, sunscreen use in U.S adults was only 31.5% [21]. Only 10% of Americans reported using sunscreen daily with nearly half (47%) indicating that they have never used sunscreen. This highlights the importance of behavior change community-based interventions to address the underutilization of sunscreen [21].

Previous studies utilized a range of theoretical frameworks, including a transtheoretical model, health belief model, precaution adoption model, social cognitive theory, protection motivation theory, inoculation theory, and theory of planned behaviors in guiding public health interventions targeted at reducing the risk of skin cancer by promoting sunscreen usage, and thereby decreasing sun exposure [22,23,24,25,26]. Such theoretical interventions have received some success in identifying gaps in knowledge, attitudes, and practices, but overall, their impact has been limited in promoting sunscreen usage behaviors. Additionally, public health experts and behavior change theorists have been cognizant of the limitations of public health theories, as many of these do not provide robust estimations of the likelihood of initiation and sustenance. Behavioral change is a long-term process, and if a behavior is not sustained long enough then a relapse is more likely. Therefore, it is vital to obtain a better understanding of the initiation and sustenance of sunscreen usage in the community, to reduce the increasing incidence risk of skin cancer. Sharma (2015) attempted to address these gaps by combining constructs of popular theories and models in a way that predicted the initiation and sustenance of a behavior [27]. Therefore, this study aims to investigate the predictability of adopting and continuing sunscreen usage behavior among a high UV index risk population: Florida residents.

## 2. Materials and Methods

### 2.1. Setting, Study Design, and Sample

This study is a cross-sectional study of the general population of Florida, which is the third largest state in the U.S., with a diverse demography [28]. The data for this study were collected through a web-based survey; launched on 11 June 2021 and closed on 27 June 2021. All participants were required to be 18 years or above, current residents of Florida, and able to read and write in English. No other exclusion criteria were applied.

### 2.2. Sample Recruitment

Invitations to participate in this study were sent through emails, which were randomly selected from the Florida vote file. Initial emails were sent to all potential respondents (~100 k) and a follow-up reminder email was sent after 4 days to the non-respondents. We oversampled (~41 k) to reach underrepresented populations to yield a representative sample comparable to the census distribution according to the latest American Community Survey population estimates.

### 2.3. Study Approval and Data Protection Compliance

The study (protocol #1015079-8) received an exempt status by the Institutional Review Board (IRB), University of North Florida. All participants received a detailed participant information sheet outlining the purpose of the study, exact details related to participation (associated risks and benefits), and how information would be stored and disseminated as one or another form of scholarly product. Participants were also informed about their voluntary participation and that they could withdraw from the study at any time. Detailed contact information of the principal investigator was provided if participants had any questions about the study. Data integrity was ensured in accordance with all data privacy laws and regulations. Principal investigators shared deidentified password protected data files with the analyst of this study. The data were stored in a locked computer and analysis results were shared in an aggregate form with the rest of the research team members.

### 2.4. Quality Control and Authenticity of Responses

Several quality control measures were applied to ensure authenticity of the responses. The “Prevent Ballot Box Stuffing” option was selected in the Qualtrics to limit only one response from each participant to collect unique responses. Invalid or incomplete entries were removed.

### 2.5. Survey Instrument

**Figure 1:**
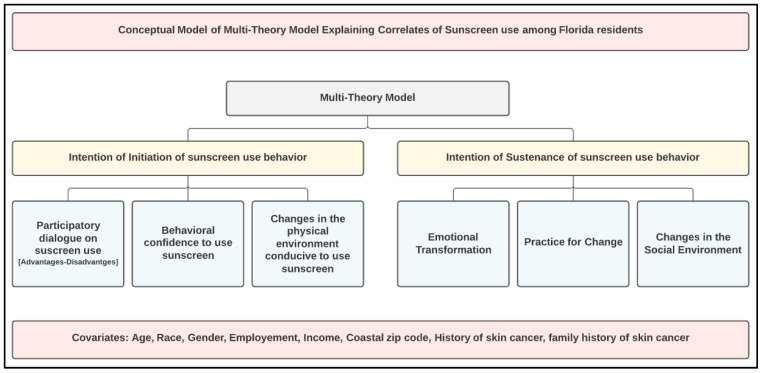
Multi-theory model framework.

The initial draft of the survey was sent to seven panelists (including three authors). All were experts in instrumentation in social and behavioral health sciences, five were experts on MTM, two were experts in sun protection research, and three were chosen from the target population. The instrument was validated for face and content validity, along with readability, in two rounds. A total of 16 changes were made during two rounds. Consensus was reached between the experts after two rounds, to finalize the survey instrument. To minimize observer bias, all reviewers were blinded. A 51-item survey MTM based questionnaire was created to examine determinants of sunscreen use among Florida residents. The Flesch reading ease of the entire scale was 66.9 and the Flesch–Kincaid grade level was 5.7 (or less than sixth grade). The survey was composed of 20 questions related to demography, outdoor activities, sunscreen use, and medical history. In addition, 31 items were related to two primary MTM theoretical constructs (initiation and sustenance). The initiation component comprised three constructs which included participatory dialogue, behavioral confidence, and changes in physical environments. “Participatory dialogue” between interventionist and subject evaluates the advantages and disadvantages of initiating an action [28,29,30]. “Behavioral confidence” is like self-efficacy but with subtle differences, it focuses on the self-confidence of the individual in acting. “Changes in physical environment” emphasizes the need for the subject to modify available resources and settings for a behavior to occur. The other component, sustenance (a continuation of behavior) comprises another three constructs: emotional transformation, practice for change, and changes in social environment. “Emotional transformation” involves changes in feelings and attitude and in this process, an individual prepares mentally to sustain the action [28,29,30]. “Practice for change” is a reflective process that continues while person is in action phase. The individual monitors behavioral progress and brings needed changes to sustain the behavior. “Changes in social environment” captures the available support around the individual that is conducive to sustaining the behavior [28,29,30]. A visual representation of MTM constructs is provided in Figure 1.

### 2.6. Statistical Analysis

Participants’ responses were first preprocessed and then exported to IBM SPSS version 27.0 (IBM Corp. Armonk, NY, USA) for statistical analyses. Incomplete responses and those with invalid data entries were excluded. Mean and standard deviation were used to represent continuous variables. Counts and proportions were used to express categorical variables. Inferential statistics were conducted through independent samples-*t*-tests to perform group-wise comparisons. Cronbach’s alpha values were computed for the entire scale and subscales to assess the internal consistency. Two hierarchical regression models (HRM) were fit to explain the variance in the likelihood of initiation and sustenance of sunscreen use behavior by MTM individual constructs, besides the demographic variables. Structural equation modeling (SEM) was utilized for the construct validation. The Analysis of Moment Structure, AMOS (Chicago, IL, USA) was used for SEM [27]. We used indices such as chi-square (χ^2^), root mean square error of approximation (RMSEA), comparative fit index (CFI), and Tucker–Lewis (TLI) to assess how well our models fit the data [31,32,33]. Models were considered to have adequate fit if they met the less stringent, but traditionally accepted, values of 0.90 or greater for CFI and TLI, and values less than 0.08 for RMSEA. *P*-values less than 0.05 were considered statistically significant.

### 2.7. Sample Size Justification

Sample size estimation of independent-samples-test was conducted using G * Power software packages, using a Cohen’s small effect size of 0.2 at the power of 95% [34,35]. After factoring a 15% attrition rate (*n* = 163), the minimum sample required was 1247 participants. For the purpose of structural equation analyses, a minimum sample size of 300 was determined to be acceptable, as indicated by previous studies [36].

## 3. Results

A total of 1284 valid responses were included in the analysis. The mean age of the sample was 50.2 ± 18.1 years. Nearly 86% participants reported living in zip codes not touching an ocean or gulf area (Table 1). About 75 percent of the sample population reported having no college level education or degree. White respondents represented nearly half of the sample population. Six out of 10 respondents reported being employed and having an annual income under USD 100,000 (Table 1). About 13 percent of respondents had a history of skin cancer. Noticeably, 4 in 10 respondents had a family history of skin cancer (Table 1). Sunscreen users had a statistically significant higher mean scores for initiation (2.10 ± 1.49 vs 0.41 ± 1.2) and sustenance (1.82 ± 1.46 vs 0.36 ± 0.74) compared to sunscreen non-users (Table 2).

Among participants who were engaged in sunscreen usage behaviors, the final model containing the demographic variables and all three constructs to predict initiation was statistically significant (adjusted R^2^ = 0.736, F = 113.572, *p* < 0.001; Table 3). In the same group, constructs of emotional transformation, practice for change, and changes in social environment (besides family history of skin cancer) were significant predictors of the sustenance of sunscreen usage behavior (adjusted R^2^ = 0.590, F = 59.565, *p* < 0.001; Table 3). Among participants who were not engaged in sunscreen usage behaviors, the model containing all constructs of initiation that were significant predictors of initiation was statistically significant; adjusted R^2^ = 0.500, F = 61.305, *p* < 0.001; Table 4. In the sustenance model, emotional transformation, practice for change, and changes in the social environment explained 23.9% of variance in sustaining sunscreen usage behaviors among those who did not engage in sunscreen usage behavior (adjusted R^2^ = 0.239, F = 19.80, *p* < 0.001; Table 4).

### Construct Validation through Structural Equation Modeling

**Figure 2:**
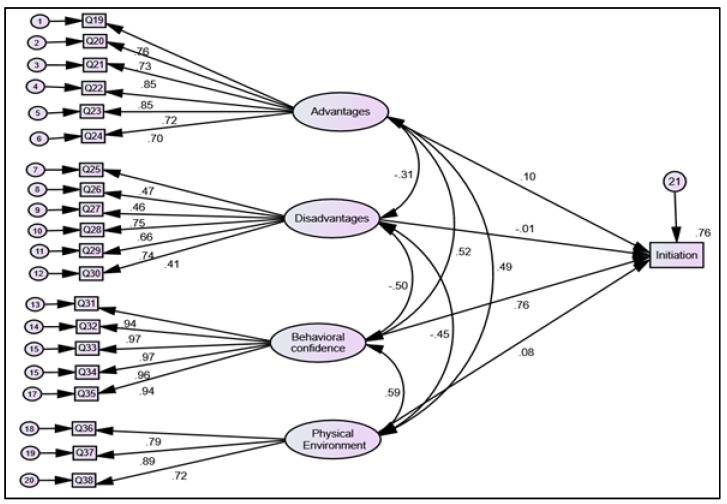
Structural equation modeling for initiation of sunscreen use behavior among Florida residents.

The structural equation modeling results (e.g., χ^2^ [252] = 1511.870 (*p* < 0.001), CFI = 0.93, TLI= 0.92, and RMSEA = 0.08) for the initiation model demonstrated the goodness of fit of the data. Standardized effects of latent variables on the factor loading indicators were observed. The factor loadings of all the subscales of initiation are shown in Figure 2.

**Figure 3:**
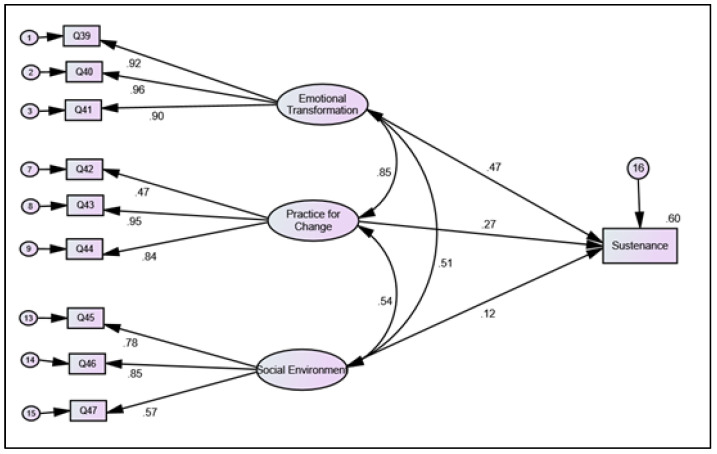
Structural equation modeling for sustenance of sunscreen use behavior among Florida residents.

The sustenance model fit the data well (e.g., χ^2^ [30] = 193.871 (*p* < 0.001), CFI = 0.98, TLI = 0.97, and RMSEA = 0.07). The factor loadings for emotional transformation, practice for change, and changes in the social environment were statistically significant. The factor loadings for all the subscales of sustenance are shown in Figure 3. The between construct correlations and standardized regression coefficients for emotional transformation showed moderate direct effects on the sustenance of sunscreen behavior, with β ranging from 0.12 to 0.51. However, both practice for change and changes in the social environment did not have any significant effects on the sustenance of sunscreen use behavior.

## 4. Discussion

The purpose of this study was to identify the correlates of sunscreen use, based on the fourth-generation multi-theory model (MTM) of health behavior change among Florida residents. MTM has been tested or applied to explain various health behaviors in community settings [37,38,39,40,41,42,43]. The results of the study were encouraging; the contribution of MTM constructs in all four models tested were significant and accounted for a substantial proportion of variance in the dependent variables. In our sample, 40.7% of the respondents used sunscreen, which was higher than the national rate of 31.5% [21]. However, sunscreen behavior was still low, given the second highest rate of skin cancer in Florida [4,5]. Understanding the determinants of sunscreen behavior is an important first step in promoting sunscreen usage behavior.

In the group who indicated sunscreen usage, all three MTM constructs (participatory dialogue, behavioral confidence, and changes in the physical environment), along with gender, race, and a history of skin cancer, were found to be significant predictors. This accounted for 73.6% of the variance in the initiation of the use of sunscreen, which is substantive in behavioral and social sciences [30]. Likewise, for sustaining sunscreen behavior among those who were already using sunscreens, all three constructs of MTM (emotional transformation, practice for change, and changes in the social environment) were found to account for 59.0% variance in the continuation of sunscreen usage. Moreover, all three constructs of MTM (participatory dialogue, behavioral confidence, and changes in the physical environment), along with gender and race, were significant explanatory variables for initiating sunscreen usage behavior among those who were not currently using sunscreen and accounted for 50% of the variance in initiation. Equally important was the finding that all three constructs of MTM (emotional transformation, practice for change, and changes in the social environment), along with gender and race, significantly accounted for 23.9% of the variance in the intention to maintain sunscreen usage behavior. These findings lend support to MTM as a strong model for designing, implementing, and evaluating sunscreen promotion interventions in the general population.

Consistently with previous studies, males were less likely to initiate use of sunscreens, both among those who were sunscreen users and those who were not. Holman and colleagues (2018), in their national study with 31,162 respondents, found that 22.1% of men compared to 40.2% of women used sunscreens [21]. Gender differences associated with intentional UV exposure through indoor tanning were also studied by previous reports [44,45,46]. In a U.S. based study, a higher proportion of females reported using indoor tanning compared to their male counterparts; however, data describing the setting (indoor or outdoor) of sunscreen use were insufficient [44,45,46]. Another interesting finding of our study was that history of skin cancer was positively associated with initiation of sunscreen usage behavior among those who were sunscreen users, but was not significant among those who did not use sunscreens. This could be explained by the reasoning that non-users were not concerned as much about their getting skin cancer or did not have “cues to action.” The MTM can play a vital role in motivating this group of non-users. This finding provides additional support for designing sunscreen promotion interventions based on MTM. Another intriguing finding was that family history of skin cancer was a positively associated significant factor for both initiation and sustenance of sunscreen usage among those who were sun screen users, indicating that users were indeed convinced of the benefits of wearing sunscreens. These findings provide the basis of developing MTM-based interventions to promote sunscreen use.

### 4.1. Strengths and Limitations

To our knowledge, this was the first study to apply MTM to explain sunscreen usage behavior. We collected data on a multitude of correlates and adjusted for those in our analysis to generate robust estimates. Despite these strengths, this study is not without limitations. First, the use of a cross-sectional design has the limitation of collecting information on independent variables and dependent variables at the same time, thereby precluding causal inferences. Future studies could test the validity of MTM in experimental designs, whereby actual manipulation of the variables is done in a longitudinal manner. Furthermore, self-reports are liable to several shortcomings, such as dishonesty, exaggerated responses, and so on. However, when measuring attitudes, one cannot choose another approach and these are indeed the only means. Finally, the study was done in Florida, thereby limiting the generalizability to other parts of the country.

### 4.2. Implications for Practice

The MTM offers a valuable prototype to design efficacious and effective sunscreen promotion interventions. Such interventions can be delivered in community settings, such as through recreational centers, faith-based organizations, community centers, community-based organizations, beach clubs, and other such venues. The promotional interventions could take the form of media campaigns; social media campaigns; one to one health education interventions, as well as in group forums; events and fairs; m-health interventions; counseling at clinics and patient care settings; and policy level efforts. For promotional interventions, the construct of participatory dialogue can be mobilized by underscoring the advantages, such as appeals to health, prevention of skin cancer, having peace of mind, not getting sunburn, and so on. At the same time, misperceptions about the disadvantages, such as staining, inconvenience, forgetfulness, cost, etc. can be clarified through discussion. The construct of behavioral confidence can be built through discussions on building assurance through self-reflection and other sources and overcoming potential barriers. The construct of changes in the physical environment can be developed through looking into the possibility of making sunscreen available to those who cannot afford it, discussing methods of application during travel and also looking into mobilizing policy support in this direction. Regarding the maintenance constructs, for emotional transformation, learning to identify feelings must be the first step in educational interventions. Then ways of converting these feelings, especially those that are negative, along with self-motivation and overcoming self-doubt must be undertaken. Regarding the practice for change construct, methods of monitoring sunscreen application behavior through apps or simple record keeping in a diary should be discussed. Troubleshooting lapses in practice and remedies must also be discussed. Finally, ways to mobilize support from family, friends, social media, health professionals, etc. must be discussed.

## 5. Conclusions

This was the first study undertaken to study the determinants of sunscreen usage behavior, using the fourth-generation, multi-theory model (MTM) of health behavior change. All the constructs of MTM were found to be significant predictors of sunscreen use among Floridians, thereby lending support to this model. All the constructs of MTM are modifiable, making it a practical approach for effecting behavior change. This study provides preliminary data to develop and test theory-based interventions to promote sunscreen usage among Florida residents.

## Figures and Tables

**Table 1:** Demographic characteristics of the sample population of Floridians (*N* = 1284).

Characteristics or Variables	n	Percentage
**Age (in years)**		
18–24	146	11.34
25–34	204	15.91
35–44	194	15.09
45–54	203	15.81
55–64	214	16.63
65 and over	324	25.23
**Gender**		
Male	619	48.24
Female	664	51.76
**Coastal Zip Code**		
Not Touching Ocean or Gulf	1104	86.02
Touches Ocean or Gulf	180	13.98
**Race**		
White	689	53.69
Black	194	15.15
Hispanic	333	25.91
Other	67	5.25
**Education**		
No College Degree	900	70.08
College Degree	371	28.90
**Employed**		
Yes	777	60.55
No	487	37.91
**Annual Income**		
Less than $ 50,000	434	33.81
$ 50,001 to $ 100,000	402	31.28
$ 100,001 to $ 150,000	185	14.39
$ 150,001 to $ 200,000	71	5.56
More than $ 200,000	89	6.95
**Skin cancer history**		
Yes	171	13.35
No	1107	86.21
**Family history of skin cancer**		
Yes	318	24.75
No	960	74.74

Note: Percentage may not add to 100%, due to some missing data.

**Table 2:** Possible and observed range and mean scores of multi-theory model constructs of behavior change across participants who engaged in sunscreen usage behavior and those who did not engage in sunscreen usage behavior (*n* = 1284).

	Groups	Participants Who Engaged in Sunscreen Usage Behavior (*n* = 523)			Participants Who Did Not Engage in Sunscreen Usage Behavior (*n* = 761)			
Constructs		Possible Score Range	Observed Score Range	Mean ± SD	Possible Score Range	Observed Score Range	Mean ± SD	*p*-Value
Initiation		0–4	0–4	2.10 ± 1.49	0–4	0–4	0.41 ± 0.80	<0.001
Participatory dialogue: advantages		0–24	0–24	15.91 ± 5.00	0–24	0–24	10.76 ± 6.30	<0.001
Participatory dialogue: disadvantages		0–24	0–22	5.75 ± 4.07	0–24	0–24	8.26 ± 5.25	<0.001
Participatory dialogue		−24 to (+24)	−16 to (+24)	10.17 ± 7.07	−24 to (+24)	−24 to (+24)	2.52 ± 8.48	<0.001
Behavior confidence		0–20	0–20	9.35 ± 6.73	0–20	0–20	1.61 ± 3.12	<0.001
Changes in the physical environment		0–12	0–12	7.43 ± 3.63	0–12	0–12	4.19 ± 3.65	<0.001
Sustenance		0–4	0–4	1.82 ± 1.46	0–4	0–4	0.36 ± 0.74	<0.001
Emotional transformation		0–12	0–12	6.73 ± 3.93	0–12	0–12	2.50 ± 3.18	<0.001
Practice for change		0–12	0–12	4.65 ± 3.30	0–12	0–12	1.98 ± 2.78	<0.001
Changes in the social environment		0–12	0–12	4.48 ± 3.73	0–12	0–12	2.76 ± 3.36	<0.001

**Table 3:** Hierarchical multiple regression (HRM) predicting likelihood of initiation and sustenance among respondents who used sunscreen (*n* = 523).

Variables	Model 1		Model 2		Model 3		Model 4	
	B	*β*	B	*β*	B	*β*	B	*β*
**The likelihood of initiation as a dependent variable**								
(Constant)	1.927		0.640		−0.224		−0.553	
Age	0.001	0.001	0.004	0.047	0.007 *	0.084	0.007 **	0.088
Gender (Female Ref.)	−0.445 **	−0.147	−0.376 **	−0.124	−0.243 **	−0.081	−0.205 *	−0.068
Race (White Ref.)	0.696 **	0.232	0.531 **	0.177	0.480 **	0.160	0.602 **	0.201
Employment (Not working Ref.)	−0.065	−0.021	−0.014	−0.005	−0.001	0.000	−0.007	−0.002
Annual Income (<$100,000 Ref.)	−0.016	−0.005	−0.008	−0.003	−0.128	−0.042	−0.152	−0.050
Education (No college degree Ref.)	−0.006	−0.002	−0.107	−0.034	0.079	0.025	0.017	0.005
Coastal Zip Code (Not touch ocean Ref.)	0.185	0.042	0.088	0.020	0.100	0.023	0.092	0.021
Skin Cancer (No Ref.)	0.052	0.013	−0.045	−0.011	−0.184	−0.045	−0.195	−0.048
Family History of Skin Cancer (No Ref.)	0.364 *	0.109	0.269 **	0.081	0.288 **	0.087	0.278 **	0.083
Participatory dialogue	-	-	0.117 **	0.552	0.026 **	0.123	0.013 *	0.062
Behavioral confidence	-	-	-	-	0.166 **	0.746	0.141 **	0.636
Changes in the physical environment	-	-	-	-	-	-	0.086 **	0.210
R^2^	0.062	-	0.360	-	0.724	-	0.742	-
F	3.517 **	-	26.758 **	-	112.985 **	-	113.572 **	-
Δ R^2^	0.062	-	0.298	-	0.364	-	0.018	-
Δ F	3.517 **	-	221.294 **	-	624.447 **	-	33.888 **	-
**The likelihood of sustenance as a dependent variable**								
Constant	1.924	-	−0.165	-	−0.274	-	−0.392	-
Age	−0.008	−0.094	−0.002	−0.023	−0.001	−0.014	0.000	−0.004
Gender (Female Referent)	−0.382 *	−0.128	−0.115	−0.039	−0.101	−0.034	−0.114	−0.038
Race (White Ref.)	0.539 **	0.182	0.486 **	0.165	0.508 **	0.172	0.493	0.167
Employment (Not working Ref.)	0.124	0.041	0.099	0.033	0.042	0.014	0.088	0.029
Annual Income (<$100,000 Ref.)	0.013	0.004	−0.074	−0.025	−0.048	−0.016	−0.054	−0.018
Education (No college degree Ref.)	−0.060	−0.019	−0.041	−0.013	−0.061	−0.019	0.006	0.002
Coastal Zip Code (Not touching ocean Ref.)	0.224	0.051	0.034	0.008	0.052	0.012	0.029	0.007
Skin Cancer (No Ref.)	0.125	0.031	0.045	0.011	0.047	0.012	0.036	0.009
Family History of Skin Cancer (No Ref.)	0.286	0.087	0.223 *	0.068	0.246 **	0.075	0.216 *	0.066
Emotional transformation	-	-	0.265 **	0.709	0.201 **	0.538	0.181 **	0.486
Practice for change	-	-	-	-	0.110 **	0.249	0.094 **	0.211
Changes in the social environment	-	-	-	-	-	-	0.058 **	0.148
R^2^	0.068	-	0.554	-	0.586	-	0.600	-
F	3.902 **	-	59.502 **	-	61.344 **	-	59.565 **	-
Δ R^2^	0.068	-	0.486	-	0.031	-	0.014	-
Δ F	3.902 **	-	521.764 **	-	36.102 **	-	17.157 **	-

B (Unstandardized coefficient); β (Standardized coefficient), * *p*-value < 0.05; ** *p*-value < 0.001; Adjusted R^2^ of initiation = 0.736; Adjusted R^2^ of sustenance = 0.590.

**Table 4:** Hierarchical Multiple Regression (HRM) predicting likelihood of initiation and sustenance among respondents who did not use sunscreen (*n* = 761).

Variables	Model 1		Model 2		Model 3		Model 4	
	B	*β*	B	*β*	B	*β*	B	*β*
**The likelihood of initiation as a dependent variable**								
(Constant)	0.474	-	0.429	-	0.115	-	0.019	-
Age	−0.001	−0.023	−0.001	−0.020	0.000	−0.009	0.000	−0.008
Gender (Female Referent)	−0.256 **	−0.158	−0.238 **	−0.147	−0.113 *	−0.070	−0.117 *	−0.072
Race (White Ref.)	0.130	0.081	0.102	0.063	0.091	0.056	0.112 *	0.069
Employment (Not working Ref.)	0.030	0.018	0.009	0.005	0.057	0.034	0.052	0.031
Annual Income (<$100,000 Ref.)	0.070	0.040	0.044	0.025	0.008	0.004	−0.009	−0.005
Education (No college degree Ref.)	0.080	0.044	0.041	0.023	0.012	0.007	−0.018	−0.010
Coastal Zip Code (Not touching ocean Ref.)	−0.036	−0.016	−0.089	−0.038	−0.014	−0.006	−0.011	−0.005
Skin Cancer (No Ref.)	−0.084	−0.033	−0.104	−0.041	−0.117	−0.046	−0.138	−0.054
Family History of Skin Cancer (No Ref.)	0.091	0.047	0.040	0.021	0.082	0.043	0.084	0.044
Participatory dialogue	-	-	0.037 **	0.394	0.016 **	0.174	0.013 **	0.143
Behavioral confidence	-	-	-	-	0.154 **	0.601	0.146 **	0.568
Changes in the physical environment	-	-	-	-	-	-	0.029 **	0.133
R^2^	0.038	-	0.190	-	0.494	-	0.508	-
F	3.129 **	-	16.768 **	-	63.323 **	-	61.305 **	-
Δ R^2^	0.038	-	0.152	-	0.304	-	0.014	-
Δ F	3.129 **	-	134.267 **	-	428.455 **	-	20.275 **	-
**The likelihood of sustenance as a dependent variable**								
Constant	0.218	-	−0.091	-	−0.087	-	−0.139	-
Age	0.001	0.034	0.002	0.054	0.002	0.052	0.003	0.065
Gender (Female Referent)	−0.190 **	−0.127	−0.123 *	−0.082	−0.135 *	−0.090	−0.147 **	−0.098
Race (White Ref.)	0.192 **	0.129	0.143 *	0.096	0.132 *	0.088	0.137 *	0.092
Employment (Not working Ref.)	0.072	0.047	0.081	0.052	0.065	0.042	0.073	0.048
Annual Income (< $100,000 Ref.)	−9.921	0.000	−0.040	−0.025	−0.013	−0.008	−0.029	−0.018
Education (No college degree Ref.)	0.058	0.035	0.033	0.020	0.039	0.023	0.042	0.025
Coastal Zip Code (Not touching ocean Ref.)	0.064	0.030	0.086	0.040	0.089	0.042	0.087	0.041
Skin cancer (No Ref.)	−0.084	−0.036	−0.106	−0.045	−0.117	−0.050	−0.111	−0.047
Family History of skin Cancer (No Ref.)	0.132	0.074	0.129 *	0.072	0.129 *	0.072	0.129 *	0.072
Emotional transformation	-	-	0.107 **	0.455	0.084 **	0.358	0.080 **	0.340
Practice for change	-	-	-	-	0.036 *	0.135	0.028 *	0.107
Changes in the social environment	-	-	-	-	-	-	0.019 *	0.085
R^2^	0.036	-	0.239	-	0.247	-	0.252	-
F	2.927 **	-	22.143 **	-	21.028 **	-	19.800 **	-
Δ R^2^	0.036	-	0.0203	-	0.008	-	0.005	-
Δ F	2.927 **	-	188.128 **	-	7.758 *	-	4.980 *	-

B (Unstandardized coefficient); β (Standardized coefficient), * *p*-value < 0.05; ** *p*-value < 0.001; Adjusted R^2^ of initiation = 0.500; Adjusted R^2^ of sustenance = 0.239.

## Data Availability

The data presented in this study are available on request from the corresponding author. The data are not publicly available due to ethical reasons.
